# New insights into risk variables associated with gas embolism in loggerhead sea turtles (*Caretta caretta*) caught in trawls and gillnets

**DOI:** 10.1093/conphys/coad048

**Published:** 2023-07-07

**Authors:** Daniel Garcia-Parraga, Jose Luis Crespo-Picazo, Blair Sterba-Boatwright, Vicente Marco, Marta Muñoz-Baquero, Nathan J Robinson, Brian Stacy, Andreas Fahlman

**Affiliations:** Fundación Oceanogràfic de la Comunitat Valenciana, Gran Vía Marqués del Turia 19, 46005 Valencia, Spain; Fundación Oceanogràfic de la Comunitat Valenciana, Gran Vía Marqués del Turia 19, 46005 Valencia, Spain; Texas A & M University-Corpus Christi, 6300 Ocean Drive, Corpus Christi, TX 78412, USA; Fundación Oceanogràfic de la Comunitat Valenciana, Gran Vía Marqués del Turia 19, 46005 Valencia, Spain; Fundación Oceanogràfic de la Comunitat Valenciana, Gran Vía Marqués del Turia 19, 46005 Valencia, Spain; Fundación Oceanogràfic de la Comunitat Valenciana, Gran Vía Marqués del Turia 19, 46005 Valencia, Spain; Institut de Ciències del Mar, Spanish National Research Council - Consejo Superior de Investigaciones Científicas, Barcelona 08003, Spain; National Oceanic and Atmospheric Administration, National Marine Fisheries Service, Office of Protected Resources, University of Florida (Duty Station), PO Box 110885, 2187 Mowry Road, Gainesville, FL 32611, USA; Fundación Oceanogràfic de la Comunitat Valenciana, Gran Vía Marqués del Turia 19, 46005 Valencia, Spain; Kolmården Wildlife Park, Kolmården, Sweden; Global Diving Research SL, Valencia 46004, Spain

**Keywords:** Bends, breath-hold diving, decompression sickness, diving physiology, fisheries interaction, hyperbaric research

## Abstract

Tissue and blood gas embolism (GE) associated with fisheries bycatch are likely a widespread, yet underestimated, cause of sea turtle mortality. Here, we evaluated risk factors associated with tissue and blood GE in loggerhead turtles caught incidentally by trawl and gillnet fisheries on the Valencian coastline of Spain. Of 413 turtles (303 caught by trawl, 110 by gillnet fisheries), 54% (*n* = 222) exhibited GE. For sea turtles caught in trawls, the probability and severity of GE increased with trawl depth and turtle body mass. In addition, trawl depth and the GE score together explained the probability of mortality (P[mortality]) following recompression therapy. Specifically, a turtle with a GE score of 3 caught in a trawl deployed at 110 m had a P[mortality] of ~50%. For turtles caught in gillnets, no risk variables were significantly correlated with either the P[GE] or GE score. However, gillnet depth or GE score, separately, explained P[mortality], and a turtle caught at 45 m or with a GE score between 3 and 4 had a P[mortality] of 50%. Differences in the fishery characteristics precluded direct comparison of GE risk and mortality between these gear types. Although P[mortality] is expected to be significantly higher in untreated turtles released at sea, our findings can improve estimates of sea turtle mortality associated with trawls and gillnets, and help guide associate conservation efforts.

## Teaser

Depth and body mass correlate positively with intravascular and tissue gas formation and mortality following decompression in loggerhead turtles incidentally captured in trawl nets. These data provide a better understanding of the effects of fisheries interaction on sea turtles and can help reduce associated harmful effects.

## Introduction

Until 2014 (García-Párraga *et al.,* 2014), it was assumed that wild sea turtles did not suffer diving-related trauma from gas embolism (GE). It was therefore surprising when GE, similar to those associated in human divers with decompression sickness (DCS), were reported in loggerhead sea turtles (*Caretta caretta*) bycaught in gillnet and trawl fisheries (García-Párraga *et al.,* 2014). Since then, GE has also been observed in olive ridley (*Lepidochelys olivacea*), Kemp’s ridley (*Lepidochelys kempii*), green (*Chelonia mydas*) and leatherback (*Dermochelys coriacea*) sea turtles (Crespo-Picazo *et al.,* 2020; Harms *et al.,* 2020; Parga *et al.,* 2020). GE and associated clinical signs resolve with recompression therapy, providing further evidence of DCS in sea turtles (García-Párraga *et al.,* 2014; Mahon and Regis, 2014).

Turtles with moderate to severe GE have a high probability of mortality (P[mortality]) unless hyperbaric oxygen treatment is administered (García-Párraga *et al.,* 2014; García-Párraga and Crespo-Picazo, 2019a). In addition, some turtles initially appear asymptomatic upon capture, but develop severe GE after being released. In field studies, 43% of turtles with GE died within 2 hours of capture by trawl fisheries and an additional 20% died within 6 days (Parga *et al.,* 2020). Delayed post-release mortality could lead to considerable underestimation of fisheries bycatch-associated mortality. Thus, there is a need to improve our knowledge of how biotic (e.g. species-specific physiology, body size, health status) and abiotic factors (e.g. fishing depth, water temperature, fishing method) alter the probability of GE (P[GE]) and associated mortality in bycaught turtles (Fahlman *et al.,* 2017; Parga *et al.,* 2017). Preliminary work on this subject has revealed that capture depth is a key risk variable associated with the P[GE] and gas bubble density based on diagnostic imaging (GE score) in loggerhead turtles (Fahlman *et al.,* 2017). Uptake and removal of N_2_, the gas most often associated with diving-related GE, are perfusion limited (Farhi, 1967). Therefore, cardiac output significantly alters the distribution of N_2_ while diving. As cardiac output allometrically scales with body size, it is also likely that body mass (*M*_b_) is a useful proxy influencing the risk of GE (Fahlman, 2017; He *et al.,* 2023). In our previous study, however, we did not have a sufficient sample size to effectively assess the impact of *M*_b_ on P[GE] and GE score (Fahlman *et al.,* 2017).

Here, we expand upon a previous analysis of risk factors associated with GE in loggerhead turtles caught as bycatch in gillnets and trawls (Fahlman *et al.,* 2017) by using a larger dataset. As in our previous study, we examined the effect of capture depth (pressure), sea surface temperature (SST) and duration that the fishing gear was in the water (soak time, a proxy for dive duration). In addition, we examined the effect of body size, a variable known to correlate with DCS in other taxa (Fahlman, 2017), with risk of GE occurrence, severity of GE and risk of mortality.

## Material and Methods

Loggerhead turtles incidentally captured in trawls or gillnets along the east coast of Spain were transported for veterinary evaluation and treatment to the ARCA del Mar, a rehabilitation facility housed at the Oceanogràfic (Table 1). All turtles were brought to the ARCA even when the animal did not show any visual signs of injury or trauma. Turtles were transported to the ARCA on the same day as their capture; however, the exact time from animal capture to arrival at the veterinary clinic was not known in all cases. The estimated depth and soak time of fishing gear was provided verbally by fishers when the turtle was handed over to the veterinary team. SST was estimated using the reported GPS position and data available on http://www.seatemperature.org/europe/spain/.

**Table 1:** **.** Numbers of examined bycaught sea turtles by gear type, average (± s.d, with range in parenthesis) curved carapace length (*CC*_L_), body mass (*M*_b_), SST, depth and estimated gear deployment time (with standard deviation and range). Superscripted values are number of animals sampled

Gear Type	*n*	*CC*_L_ (cm)	*M*_b_ (kg)	SST (°C)	Depth (m)	Soak time (hrs)
Gillnet	112	39 ± 8^106^ (22–65)	8.3 ± 6.4^96^ (1.8–33.0)	17 ± 3^99^ (13–25)	13.9 ± 6.9^73^ (3–45)	11.7 ± 4.3^56^ (1.3–24.0)
Trawl	302	43 ± 18^279^ (20–86)	13.9 ± 13.5^286^ (1.9–85.5)	15 ± 2^264^ (13–25)	47.2 ± 19.6^219^ (13–122)	2.6 ± 1.2^163^ (0.3–8.0)

On arriving at the ARCA, turtles were weighed (*M*_b_, kg) and their curved carapace length (*CC*_L_, cm) was measured from the mid-point of the nuchal scute to the posterior-most tip of the carapace (Wyneken, 2001). Turtles were also evaluated visually and by neurological examination, routine hematology and blood chemistry analysis, ultrasonography of the coelomic cavity through standard acoustic windows (General Electric Logiq E Vet ultrasound machine, GE Medical Systems), full-body radiographs in cranial-caudal (CC), lateral-lateral (LL) and dorsal-ventral (DV) projections (Philips Practix 400 unit, Philips Medical Systems; and a Kodak Direct View Classic CR System, Carestream Health), and, in select cases, computer tomography (CT) (Siemens ® Somatom Volume Zoom CT Scanner).

### GE score and treatment

In addition to the methods mentioned in the previous section, ultrasonography and radiography were used to assess the presence (1) or absence (0) of GE, and to score the severity of bubble density on a scale from 0 (no bubbles), to 5 (most severe), as previously detailed (Fahlman *et al.,* 2017). Descriptive statistics for parameters examined using our analyses are provided in Table 2.

**Table 2:** Number of turtles with GE score on a range from 1 to 5 for the different fisheries gear types. The number in superscript is the number of turtles that arrived alive with a specific GE score and later died. Subscripted numbers are number of turtles that died within each GE score

Gear type	GE Score					
	0	1	2	3	4	5
Gillnet	75	17^1^	14^1^	4^1^	0	0
Trawl	116	67	66^4^	46^5^	5	3^1^

Animals with GE were immediately treated with Hyperbaric Oxygen Therapy (HBOT) for 10–16 h at 2.6 ATA (García-Párraga and Crespo-Picazo, 2019b). All activities related to veterinary evaluation of bycaught turtles were conducted under an official signed agreement provided by the Government of the Valencia Region. The objective of the veterinary activities was to provide appropriate care and maximize survivorship, and no procedures were conducted solely for research purposes.

### Statistical analysis

When conducting statistical analyses, we removed all animals that had aspirated sea water during capture. Aspiration of sea water likely increases the risk of mortality through asphyxiation. In addition, water aspiration likely inhibits respiration and gas exchange, which in turn would affect the capacity to remove N_2_ during ascent or at the surface. While filtering reduced the sample size of the data set, this approach allowed us to avoid confounding variables and specifically examine the effects of decompression resulting from fisheries interaction.

Ordinary least squares (function *glm* or *nls* in R) were used to assess the relationship between continuous predictive variables, while we used logistic regression with the logit link (function *glm* with family binomial) to analyze binary dependent variables, e.g. presence or absence of GE. We used an exhaustive search for predictive parameters, where variables with marginal p-values < 0.05 were considered in a multivariate model. To compare nested models, we excluded those observations with missing data from either model. In addition, both the p-value of the chi-squared significance of the difference in residual deviances between the models, and the Akaike Information Criteria (AIC) were used to select models (see Statistical Appendix Sections 2.1, 2.2, 3.1 and 3.2 for further information). Finally, a Hosmer-Lemeshow (HL) goodness of fit test was used to assess the goodness of fit for the final models (Hosmer Jr. *et al.,* 2013). We also used the area under the curve (AUC) of the receiver-operating curve as an alternative measure of goodness of fit, where values > 0.7 were considered acceptable (Melo, 2013). Cardiac output is known to affect the risk of GE (Fahlman, 2017), and as *M*_b_ scales allometrically with cardiac output (He *et al.,* 2023), we used the log_10_-transformed *M*_b_ (log_10_[*M*_b_]) as a proxy for cardiac output. Because carapace length is more often available for bycaught turtles than *M*_b_, we also used *CC_L_* as an alternative to *M*_b_ to predict risk of GE or mortality.

Ordinal regression (function *clm* in R with the logistic link using proportional odds) was used to separate the risk of DCS based on GE scores. The analysis was done in a similar manner as for the logistic regression (see Statistical Appendix Sections 2.3, 2.4, 3.3 and 3.4), and the Lipsitz test was used to assess goodness of fit (Lipsitz *et al.,* 1996).

*P* ≤ 0.05 and ≤ 0.01 were considered significant and highly significant, respectively, and *P* ≤ 0.1 were considered a trend. Models were also judged based on the goodness of fit tests. Data are presented as the mean ± standard deviation (s.d.), unless otherwise stated. All statistical calculations were performed using the statistical language R (full references and code for all R tools may be found in the Statistical Appendix, see link in Data availability).

## Results

Our dataset consisted of 494 incidentally caught loggerhead turtles. Of these, we excluded 78 that had aspirated water (59 of which had GE), and three due to missing or inconsistent data related to GE observations or biometrics. Of the remaining 413 turtles, 303 were caught by trawl and 110 by gillnet (Table 2). The average duration that fishing gear was in the water (*glm*, soak time, χ^2^ = 87.7, df = 1, *P* < 0.01), and the SST (χ^2^ = 52.7, df = 1, *P* < 0.01) were significantly lower for trawls than gillnets (Table 1). In contrast, the *M*_b_ and depth of the fishing gear was greater for trawls than gillnets (*M*_b_: χ^2^ = 15.4, df = 1, *P* < 0.01; depth: χ^2^ = 12.1, df = 1, *P* < 0.01, Table 2).

The overall incidence of GE was 54% (222/413). Fourteen percent (58/413) of the study population had a GE score ≥ 3, which is typically fatal without treatment (García-Párraga *et al.,* 2014), and 93% (54/58) of those were from trawl fisheries. The deepest depth of gear deployment that resulted in a capture was 122 m for a turtle in the trawl fishery that arrived with a GE score of 5 (Table 2). Thirty-five turtles (35/110) in the gillnet group had GE, with the highest GE score of 3 observed in four turtles (Table 2). As all animals with a GE score > 0 received treatment, we were unable to estimate mortality for untreated animals. As such, we used mortality rates of untreated turtles from other studies as a comparison of the P[mortality] in treated and untreated turtles (Crespo Picazo, 2020; Parga *et al.,* 2020; Franchini *et al.,* 2021). In the trawl group, seven turtles died despite receiving recompression treatment and three arrived dead, and of those, two did not have data for *M*_b_, and one did not have information about the depth of the fishing gear. For the gillnet fishery, three turtles were dead upon arrival, but only two had information about both depth and *M*_b_.

Due to significant differences in depth and duration of gear deployment between trawl and gillnet fisheries, we analyzed turtles separately based on the method of capture.

### Trawl data

#### Probability of gas emboli

**Figure 1:**
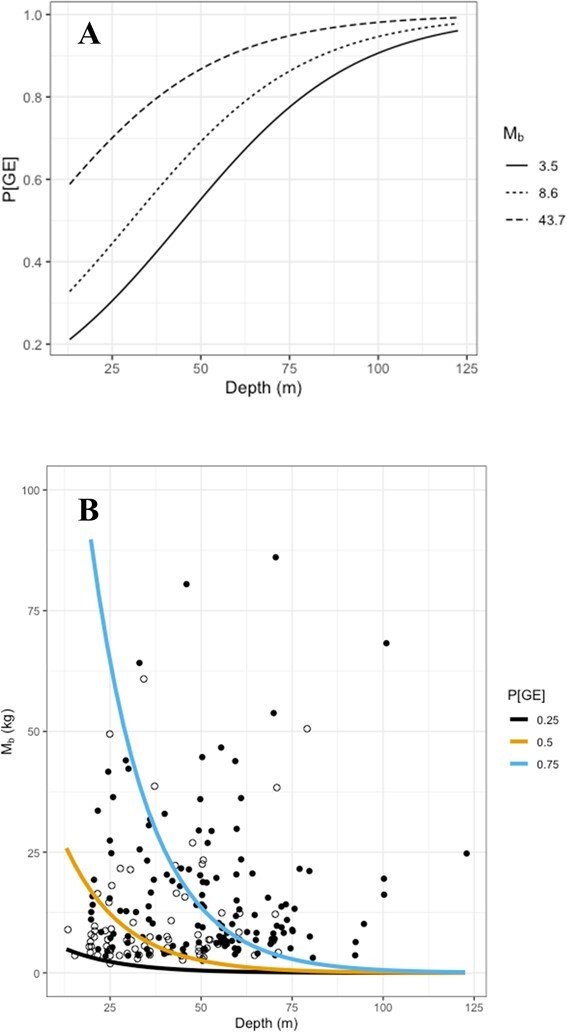
Predicted probability of gas embolism (P[GE],%) against depth (m) for trawl using depth and body mass (*M*_b_, kg) as predictors. (A) The risk for three loggerhead turtles with varying *M*_b_ in kg. Body masses used were the 5th, 50th and 95th percentile of the body masses in the data set. (B) The depth and *M*_b_, with closed and open circles for individual turtles with or without GE, and isopleths for the 25%, 50% and 75% probability of GE.

**Figure 2:**
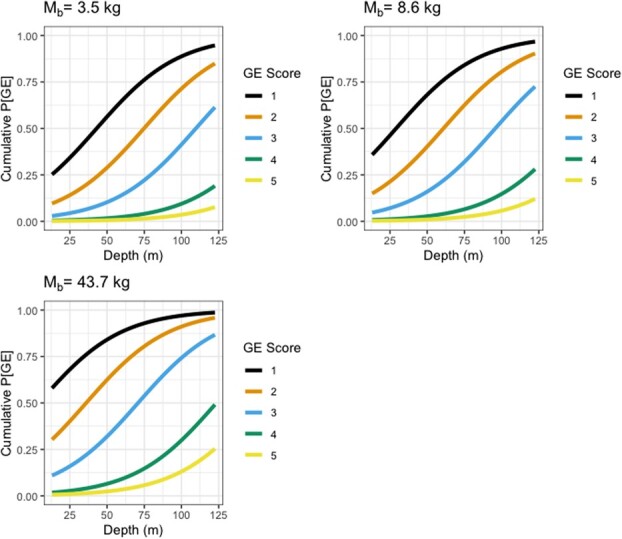
Plot of the predicted cumulative probability of gas embolism (P[GE]) for trawl fishery for a specific gas embolism (GE) score (0–5) using depth and body mass (*M*_b_) as predictive variables (Eq. 2A). Body masses used were the 5th, 50th and 95 percentile of the body masses in the data set.

For trawl caught turtles, the most parsimonious multivariate logistic regression model included log_10_[*M*_b_] (mass in kg) and depth (m) as variables that explained P[GE] (Fig. 2; Table 2 in Statistical Appendix Section 2.2.1). A combination of *CC_L_* (cm) and depth were also significant, but with an AIC that was slightly higher than the model with depth and *M*_b_ (Table 2, in Statistical Appendix Section 2.2.1). We include* CC_L_* in the Statistical Appendix, as it may be more useful in the field where a scale may not be available. The analysis indicated that increased depth, *M_b_* and *CC_L_* increased the risk of P[GE] (Fig. 1, and see Statistical Appendix Section 2.2.4).

Figure 4 in the Statistical Appendix compares the two models using either *M*_b_ or *CC_L_*, in addition to depth to predict P[GE].

We used ordinal regression to determine the risk factors for a specific GE score. For trawl, depth, length, soaking time, and log_10_[*M*_b_] were considered, and the most parsimonious model included depth and log_10_[*M*_b_] (Fig. 2; see also Statistical Appendix Section 2.3).

#### Probability of mortality for animals in rehabilitation

**Figure 3:**
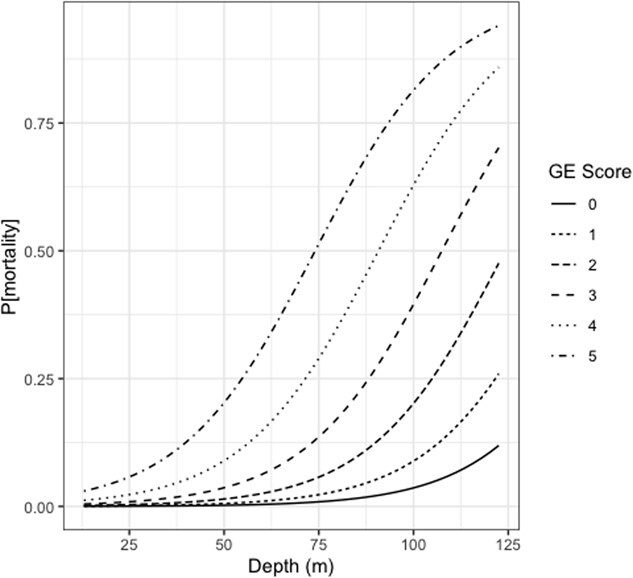
Predicted risk of mortality(P[mortality]) against depth (m) for trawl for different GE scores.

The probability of mortality for recompression treated turtles caught by trawl used depth, log_10_[*M*_b_], and GE score (as a continuous variable). For turtles with GE scores of 2, 3, and 5 the observed mortalities were, respectively, 6%, 11% and 33%. The model, including the seven turtles which had no missing data, where each variable had a marginal p-value < 0.05 contained GE score and depth (Fig. 3; Section 2.4 in the Statistical Appendix). The goodness of fit for this model had an AUC of 0.926, but as there were only seven cases with data for both depth and GE score, the HL test was unreliable (see Statistical Appendix Section 2.4.2).

### Gillnet data

#### P[GE] or GE score

For gillnet caught turtles, neither logistic nor ordinal regression found that any of the explanatory variables had the ability to significantly predict P[GE] nor GE score (see Statistical Appendix section 3.1–3.3).

#### Probability of mortality for animals in rehabilitation

The observed P[mortality] for recompression treated turtles with GE scores of 1, 2 and 3 were, respectively, 6%, 7% and 25%. The model, including the two turtles that had no missing data, where each variable had a marginal p-value < 0.05 contained GE score (as a categorial variable) and depth (see Statistical Appendix section 3.4).

As there were only two cases with data for both depth and GE score, and judging by the large size of the coefficients, this model was deemed unreliable. Additional data should be collected in future studies to improve the model reliability.

## Discussion

We show that the probability and severity of GE in loggerhead turtles caught by trawl fisheries increases with trawling depth and turtle body mass. In contrast, no predictive variable was found that significantly explained P[GE] or GE score in gillnet fisheries. Finally, for both fisheries, both GE score and depth were important to predict the risk of mortality, even when recompression therapy was administered. These results provide further evidence that mortality rates associated with bycatch may be significantly underestimated if the risks associated with GE are not considered.

### Possible risk factors for fisheries related GE

It is known that GE score can vary both between (human or animal) and within individuals that are performing the same compression and decompression sequence, i.e. dive profile, and also when the same dive profile is repeated on different days (Fahlman, 2017). Thus, each dive profile has a certain risk, or probability, of GE score or mortality. In addition to dive profile, there are several potential risk factors, such as *M*_b_, and water temperature, that may alter the probability. A better understanding of these risk factors and their effect of the risk may provide potential ways to reduce GE and its associated mortality.

In a similar study with a smaller data set (Fahlman *et al.,* 2017), we showed that depth was an important predictor for P[GE] for trawl-caught turtles but we did not have sufficient data to assess the impact of other potential risk factors such as: soak time, SST, *CC_L_* and* M*_b_ (as log_10_[*M*_b_]). Nevertheless, in the updated model, only *M*_b_ (*CC_L_*, and depth significantly predicted P[GE] and GE score, while neither SST nor soak time warranted inclusion in any model. While log_10_[*M*_b_] had a slightly better AIC as compared with *CC*_*L*_ to predict P[GE], the latter appears to be a useful proxy in a field situation where access to a scale may not be available.

It was interesting that SST did not warrant inclusion in the model. In mammals, blood flow is an important predictor of GE risk and mortality (Fahlman, 2017). As the level of blood flow in ectotherms, such as sea turtles, is tied to metabolic rate and this is, in turn, is linked to ambient temperature, it is a reasonable to hypothesize that SST influences GE risk. In the current data set, it is possible that there was not sufficient variation in the SST data to detect an effect. Indeed, most turtles were captured in the winter when the SSTs remain both cold and relatively stable. It should also be noted that SST does not correlate exactly with sea turtle body-temperatures (Sato *et al.,* 1995) and water temperatures will vary with depth; therefore, SST may not reflect animal temperature. Unfortunately, the data necessary to explore temperature more precisely upon capture were unavailable for this study population.

It is also well established that for diving animals, dive duration and P[GE] are positively related until the body is equilibrated at the new pressure, i.e. the tissues reach a gas tension equivalent to ambient pressure (Fahlman, 2017; Robinson *et al.,* 2021). However, like past studies, soak time did not appear to alter P[GE] or GE score for either trawl or gillnet fisheries. This contradicts the findings of a similar study that reported soak time was longer in turtles bycaught in trawls with GE than those without GE (Franchini *et al.,* 2021). There are a few considerations that may account for this disparity. It is possible that our data set does not have sufficient variation in soak time/gear deployment to detect an effect for either trawl or gill net captures. In addition, the exact time of capture and duration of submergence is not known and may be variable among turtles caught in gear deployed for similar intervals, which could mask an effect. Lastly, it is possible that underwater capture elicits a stress response, resulting in increased blood flow and gas uptake, which results in saturation within most submergence durations experienced by bycaught sea turtles.

In a previous study, it was shown that acetylcholine, and serotonin, resulted in contraction of the arterial pulmonary sphincter in loggerhead sea turtles, while adrenaline relaxed the sphincter (García-Párraga *et al.,* 2018a). The authors suggested that the dive response results in contraction of this sphincter and helps re-circulate blood without passing by the lung. This helps reduce N_2_ uptake during long and deep dives, but also limits exchange of O_2_ and CO_2_. However, slight relaxation of the sphincter during a dive allows a small perfusion/ventilation ratio, which, due to the large differences in solubility between O_2_, CO_2_ and N_2_, allows uptake of O_2_ and removal of CO_2_ without significant exchange of N_2_. Thus, the 3 chambered heart of the sea turtle and arterial sphincter provides a physiological mechanism that helps prevent significant build-up of N_2_ during normal diving. As stress relaxes the sphincter and increases pulmonary perfusion, this likely results in increased N_2_ uptake and GE formation during ascent (García-Párraga *et al.,* 2018b).

Interestingly, ascent rate has been proposed to be an important predictor of GE and mortality in a study of loggerhead turtles bycaught in trawls (Franchini *et al.,* 2021). It has even been hypothesized to be a key factor controlling the risk of GE in free-diving turtles under natural conditions (Fossette *et al.,* 2010; Robinson *et al.,* 2021). Similar to dive duration, or duration of submergence in captured animals, it is well established that variation in ascent rate has a large effect on P[GE] and mortality in mammals, and it has been shown that removal of as little as 5% of the inert gas burden may reduce risk by as much as 45% (Fahlman *et al.,* 2001; Lillo *et al.,* 2002; Fahlman, 2017). In the current study, we had no data on the ascent rate as this variable is difficult to reliably obtain from fishers. Nonetheless, methods of gear retrieval warrant further consideration as potential opportunities to mitigate GE.

As previously mentioned, the data from the gillnet fishery did not indicate that any predictive variable warranted consideration. Although the data consisted of 110 cases, most of the 35 cases of turtles with GE had a GE score of 1, and only 4 with a GE score of 3. In addition, the data from the gillnet fishery was limited in the depth range, with only 3 animals retrieved from depths deeper than 20 m (specifically: 40, 50 and 70 m). Of those, the turtles from 40 m and 50 m had a GE score of 1 and only the turtle caught at 70 m had a GE score of 3.

The soak time differed greatly between the trawl and gillnet fisheries, but despite the much longer soak times relative to trawl tow times, gillnets were not associated with a greater risk of GE. One difference between these two fishing modes is that trawling in Valencia region involves a net deployed at an average depth of 47 m that is constantly moving, whereas gillnets are static and set at an average depth of 14 m. Capture in these two situations may have considerable differences in the resulting stress response, intensity of physical activity and associated physiological changes, and increase in cardiac output. Also, the turtles captured by gillnet were smaller, which according to our data from turtles bycaught in trawls reduces the P[GE] and the probability of a higher GE score. Another plausible explanation for our observations is that submergence for 2.6 hours, the average deployment of trawls in the Valencian region, results in forced submersions long enough to reach gas equilibrium and therefore longer submergence durations, as in the gillnet fishery, do not alter P[GE] or mortality from GE; however, longer soak time may result in higher prevalence of water aspiration and asphyxia (Snoddy and Williard, 2010). Thus, these confounding factors make it difficult to draw firm conclusions about potential differences in GE risk between trawl and gillnet. In addition, the current study confirms that for the trawl fishery the risk of GE increases with depth, but that the P[GE] also increases with increasing *M*_b_.

### Predicting risk of mortality following treatment based on depth and GE score

In DCS research in terrestrial mammals, it is still equivocal whether higher bubble grade, i.e. GE score, results in greater risk of DCS symptoms (Nishi *et al.,* 2000; Brubakk and Eftedal, 2001; Bernaldo de Quirós *et al.,* 2016; Papadopoulou *et al.,* 2018). One reason for conflicting results may be due to challenges associated with detecting mild signs of DCS, especially in small and medium sized animals. Yet mortality is an undisputable end-point, and one study in rabbits showed a clear correlation between GE score and mortality following rapid decompression (Bernaldo de Quirós *et al.,* 2016). In our previous study, we did not have sufficient data to test which variables could be used to predict the risk of mortality; therefore, we assumed that a GE score of 3 or higher resulted in a high probability of mortality based on clinical observations and predicted that the capture depth resulting in 50% mortality of bycaught turtles is around 65 m (Fahlman *et al.,* 2017). With this larger data set, we aimed to investigate whether the regression model, which included additional predictor variables for each fishery could predict the probability of mortality (Fig. 3). Although turtles that receive treatment likely have a different outcome that those released at sea, by using actual observed mortality rather than a predicted outcome (i.e. based on GE score) we were able to avoid extrapolation. The analysis showed that both increasing GE score and depth increased the risk of mortality in turtles bycaught in trawls (Eqs. 3A and B). For a GE score of 1, 3 and 5, the depth for a 50% mortality rate was, respectively, 141 m, 107 m, and 74 m. For gillnet, either GE score or depth, but not both, predicted mortality. A depth of 45 m or a GE score of between 3 and 4 resulted in 50% mortality. Although we were not able to show that depth relates to P[GE] in gillnets, as suggested for trawls, this influence of GE severity or depth on mortality requires further exploration. In addition, the steeper slope of the regression between trawl and gillnet, suggests that there are differences in mortality risk between the fisheries represented in this study. Whether those differences depend on dissimilarities in duration of submergence (i.e. length of gear deployment), water temperature at depth, *M*_b_, or depth are still unresolved.

As all turtles with GE were treated with recompression, our mortality rates should not be directly applied to scenarios involving GE without veterinary intervention (e.g. when bycaught turtles are released at-sea without treatment). Thus, these results provide a conservative estimate of the relationship between GE severity and mortality that may help to guide management. Studies conducted on board fishing vessels in the Adriatic Sea and Brazilian waters have examined GE progression and mortality rate in satellite tagged animals released directly into the ocean without recompression therapy. In one study, 54% (15 out of 28) of bycaught loggerhead turtles died, and of those 12 died within 2 hours on-board the fishing vessel and the remaining within 6 days of being released (Parga *et al.,* 2020). In another study, only 2% (9/482) of all bycaught loggerhead turtles that arrived at the rehabilitation facility were dead or later died, but of 12 turtles that arrived with severe symptoms 10 died within 24 hours (Franchini *et al.,* 2021). Finally, one study reports the probability of death as 50% (4/8), 45% (5/11), and 50% (2/4), for turtles with GE classified as severe, moderate to severe, and moderate, respectively (Crespo Picazo, 2020).

### Summary

In the current study we provided new information suggesting that both depth and *M*_b_ are important to predict the probability and severity of GE in loggerhead sea turtles bycaught in trawls. The limited *M*_b_ and depth range for turtle caught in gillnets are likely reasons why no relationships were found between the explanatory variables and gas embolism for this gear type. However, for both trawls and gillnets, we demonstrated that depth and GE severity are important for predicting risk of mortality even in sea turtles that receive recompression therapy following incidental capture in fishing gear. Thus, we should consider much higher mortality under typical conditions when animals are released untreated. We urge caution in use of these models until additional data can confirm their validity, improve their confidence limits and explore other factors of interest related to GE and DCS. This work is another example of valuable collaboration between fishers, researchers, veterinarians and local and international groups that can support conservation of wild species and also allow fisheries to become more sustainable.

## Data Availability

The Statistical Appendix, R-code and data sets used in this study are freely available at the following link: http://osf.io/5t9qg
